# A single dose of the Biontech/Pfizer BNT162b2 vaccine protected elderly residents from severe COVID‐19 during a SARS‐coronavirus‐2 outbreak in a senior citizen home in Germany

**DOI:** 10.1002/iid3.532

**Published:** 2021-09-16

**Authors:** Rolf Schwarzer, Sabine D. Freys, Nick Neuwinger, Nina Beikert, Bettina Eberspächer, Anke Edelmann, Marta Zuchowski, Inga Slothouwer, Angela Stein, Kathrin Theil, Peter Menzel, Jörg Hofmann

**Affiliations:** ^1^ Labor Berlin—Charité Vivantes GmbH Berlin Germany; ^2^ Public Health Department Bremen Bremen Germany

**Keywords:** infections, processes, vaccination, viral/retroviral

## Abstract

**Background**: A total of 62/66 (93.9%) residents in a senior citizen home in Bremen, Germany, received the first dose of the Biontech/Pfizer vaccine BNT162b2 on December 27th 2020. After routine severe acute respiratory syndrome coronavirus 2 (SARS‐CoV‐2) antigen tests showed positive results on January 5th, all residents and staff were tested by RT‐PCR.

**Results**: Nine staff members and 23 residents had a positive result. PCR positive staff members reported mild to severe COVID‐19 symptoms, one was hospitalized. None of them had been vaccinated. In contrast, the vaccinated residents reported no or only mild symptoms. Sequencing of the SARS‐CoV‐2 genomes of infected individuals revealed a monophyletic origin of the outbreak within the PANGO lineage B.1.177.86.

**Conclusions**: In summary, our data show that partial vaccination prevented severe COVID‐19 among the residents during this local SARS‐CoV‐2 outbreak, suggesting a high effectiveness of even a single vaccine dose, but also emphasize that asymptomatic individuals might still be carriers/spreaders.

## Graphical abstract

**Background**: A total of 62/66 residents in a senior citizen home in Bremen, Germany, received the first dose of the Biontech/Pfizer vaccine BNT162b2 on December 27th 2020. After routine SARS‐CoV‐2 antigen tests showed positive results on January 5th, all residents and staff were tested by RT‐PCR.

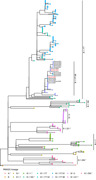

## 1 INTRODUCTION

The global pandemic caused by the emergence of the severe acute respiratory syndrome coronavirus 2 (SARS‐CoV‐2) in 2019 is still spreading in many parts of the world.^1^ While containment measures could only slow down the spread of the pandemic, effective vaccines are a key factor for preventing fatal outcomes of COVID‐19 and also have the potential to reduce viral transmission. As of early 2021, several vaccines have completed clinical trials and are currently deployed,^2^, ^3^ while many more vaccine candidates are still under development.^4^ Especially the novel class of messenger RNA based vaccines showed promising efficacy in clinical trials.^3^ Various vaccination schemes apply to the different vaccines. For the Pfizer‐Biontech COVID‐19 vaccine BNT162b2, the highest efficacy is reached 2 weeks after getting two doses of the vaccine at intervals of 3 weeks.^3^ The degree of protection after partial vaccination is the subject of many ongoing studies around the world. Especially the response of elderly people to vaccination is of interest, as, on the one hand, they have a higher risk of a severe course of COVID‐19 disease,^5^ but, on the other hand, their immune response to the vaccine might be too weak to yield full protection.^6^

## 2 MATERIALS AND METHODS

Nasopharyngeal swabs were initially tested using the cobas® SARS‐CoV‐2 test kit on Roche cobas® 6800/8800 instruments (Roche Diagnostics) in a local diagnostic laboratory and subsequently sent to our laboratory for typing and sequencing of SARS‐CoV‐2.

Serum samples were tested applying the Elecsys Anti‐SARS‐CoV‐2 assay in a cobas® e 801 system (Roche Diagnostics) for total nucleocapsid antibodies and the Anti‐SARS‐CoV‐2‐Elisa (Euroimmun Lübeck) for immunoglobulin G and immunoglobulin A to the viral spike protein according to the manufacturer's instructions.

RNA was purified using the MagNA Pure 96 DNA and Viral NA small volume kit (Roche Diagnostics) on a MagNA Pure 96 system applying the pathogen universal protocol. To estimate the viral loads in the samples to be sequenced, a SARS‐CoV‐2 specific RT‐PCR using primers targeting the E gene on a Roche Lightcycler 480 II was used according to Corman et al.^7^ RNA was transcribed into cDNA using Lunascript reverse transcriptase (New England Biolabs) according to the manufacturer's protocol. The EasySeq™ RC‐PCR SARS‐CoV‐2 WGS kit (Nimagen) was used to generate indexed sequencing ready DNA libraries as described in the manufacturer's instructions.^8^ The libraries were sequenced on an Illumina NextSeq 550 NGS system (Illumina, San Diego, California, US) using 2 × 150 bp paired‐end reads.

After sequencing, the reads were demultiplexed using *bcl2fastq* v2.20.0.422 (Illumina) with zero allowed barcode mismatches. Consensus genome sequences were generated with the Illumina workflow of the *ncov2019‐artic‐nf* pipeline (https://github.com/connor‐lab/ncov2019‐artic‐nf). Genome sequences were assigned to PANGO lineages using pangolin.^9^ (http://github.com/cov‐lineages/pangolin). To place the outbreak samples in a phylogenetic context, we downloaded all 202 SARS‐CoV‐2 genomes with collection date in January 2021 and location *Europe/Germany/Bremen* from GISAID (see supplementary information for GISAID accession numbers). Sequences were aligned using *MAFFT* v7.475^10^ and a phylogenetic tree was inferred from the alignment using *IQ‐Tree* v2.1.2.^11^ The phylogenetic tree was plotted using the R package ggtree.^12^ Columns with mutations were plotted using *snpit* (https://github.com/aineniamh/snipit).

## 3 RESULTS

As part of the national SARS‐CoV‐2 vaccination strategy in Germany, residents and staff of senior citizen homes belong to the highest prioritization group in the vaccination campaign.^13^ Hence, the residents of a senior citizen home in Bremen received their first of two doses of the Biontech/Pfizer vaccine BNT162b2 already on December 27th 2020. Before administration of the second dose, routine antigen testing on January 5th showed a putative infection in some senior residents. Subsequent RT‐PCR testing of all residents and staff revealed a SARS‐CoV‐2 outbreak in the senior citizen home. Afterwards, all residents were isolated in their rooms until the end of the outbreak. During this time, neither housemate contacts nor visitors were allowed. Staff members recorded symptoms, body temperature, oxygen saturation, and breathing rate twice a day. PCR tests were done in weekly intervals.

Positive SARS‐CoV‐2 results obtained by RT‐PCR occurred not only among unvaccinated staff members, but also among partially vaccinated residents (Table 1). Nine staff members and 23 residents had a positive PCR.

**Table 1:** Summary of data for partially vaccinated and unvaccinated residents and staff members with confirmed SARS‐CoV‐2 infections

	Partially vaccinated (*n* = 22)	Unvaccinated (*n* = 10)
Age range (y)	76–100	21–94
Sex		
female	19	8
male	3	2
Disease status		
No symptoms	20	0
Mild symptoms^a^	2	1
Severe symptoms^b^	0	9

*Note*: A confirmed case of SARS‐CoV‐2 infection was defined as a positive SARS‐CoV‐2 RT‐PCR result from respiratory material obtained from residents and staff members within 2 weeks after first vaccination.

Abbreviation: SARS‐CoV‐2, severe acute respiratory syndrome coronavirus 2.

^a^ Including tiredness, temporary lower oxygen saturation, slightly elevated body temperature

^b^ Including fever >38.5°C, dry cough, exhaustion, dyspnea, chest pain, ageusia, weakness, hospitalization, death.

Eight of the nine SARS‐CoV‐2 positive unvaccinated staff members showed severe signs of COVID‐19 (severe acute respiratory illness, one patient was shortly hospitalized). None of the vaccinated residents showed any severe signs of acute respiratory illness and had only mild symptoms. One resident had slightly elevated body temperature (up to 37.9°C), another resident was dyspneic for about 2 days, but showed no further symptoms after this short period. The only resident with severe disease signs (poor general condition, fever, supplemental oxygen necessary) was unvaccinated and died during the course of disease.

As the clinical course of infection was dramatically different between the partially vaccinated residents and the unvaccinated staff members, the question remained if an infection with different virus strains might be the cause. Therefore, patient samples with sufficient viral loads were subjected to whole genome SARS‐CoV‐2 sequencing.

In total, 16 SARS‐CoV‐2 genome sequences could be included in the analysis, 14 residents (all vaccinated) and 2 staff (both unvaccinated). Of those, 14 sequences were assigned to PANGO lineage B.1.177.86, and two sequences were assigned to the parent lineage B.1.177, which is likely a misclassification due to their higher N‐content (11.2% and 8.5%, respectively, compared to <2% in the other 14 sequences).

**Figure 1:**
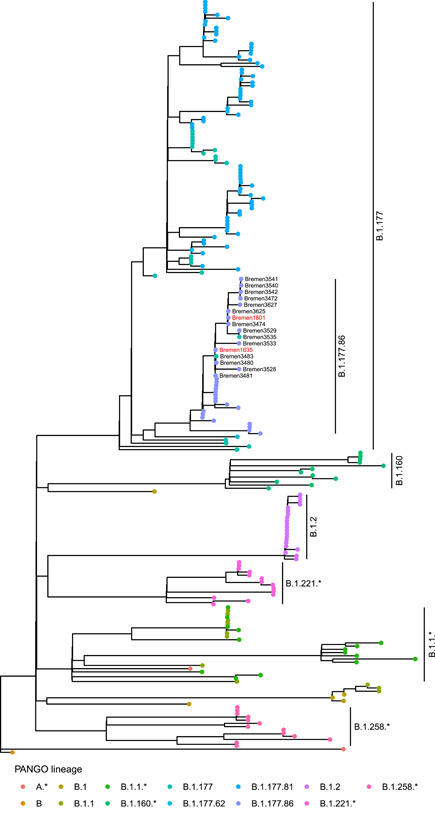
Phylogenetic tree of SARS‐CoV‐2 genomes from this study (staff members are highlighted in red) and 202 unrelated SARS‐CoV‐2 genomes downloaded from GISAID that were collected in Bremen in January 2021. Colors of dots denote the PANGO lineage, samples from this study are labeled as Bremen. SARS‐CoV‐2, severe acute respiratory syndrome coronavirus 2.

Phylogenetic analysis together with 202 unrelated samples collected in Bremen in January 2021 showed that all 16 SARS‐CoV‐2 genome sequences of residents and staff form a monophyletic group within the PANGO lineage B.177.86 (Figure 1). A comparison with the other B.1.177.86 sequences in the dataset shows that the single nucleotide variant C23533T in the S gene is uniquely observed in our samples (Figure S1).

To test for a SASR‐CoV‐2 specific immune response, serum samples were obtained 12 weeks (19th March 2021) after vaccination. Presence of antibodies against both the viral nucleocapsid and spike protein indicate an ongoing or past infection. Antibodies were detected in almost all tested individuals except one resident.

## 4 DISCUSSION

The first SARS‐CoV‐2 vaccines received their conditional market authorization for the EU only at the end of December 2020 and the global vaccine rollout will take months if not years. Therefore, studies on vaccination efficacy of the different vaccines in real‐world scenarios are crucial for determining optimal strategies for vaccination programs given limited vaccine availability.

Since elderly people are among the most vulnerable to severe COVID‐19, vaccination programs in Germany started in nursing and senior citizen homes in late December 2020. We report here on a local SARS‐CoV‐2 outbreak in a senior citizen home, affecting vaccinated residents and unvaccinated staff. Severe illness occurred only in the group of unvaccinated individuals, though. While different courses of disease could be caused by infections with strains of different virulence, genome sequencing of samples from some of the residents and staff members revealed a monophyletic origin of all analyzed samples. This finding suggests that the outbreak originated from a single transmission event.

Both vaccinated and unvaccinated individuals had RT‐PCR detectable viral loads. While our sample size is too small to compare mean Ct values between vaccinated and unvaccinated groups, previous studies have shown an age‐independent reduction of viral load after infection.^14^, ^15^, ^16^ Other studies further showed a reduction in asymptomatic infection after a single vaccine dose,^17^ whereas the observations from this outbreak rather suggest a reduction of severity in the vaccinated group. However, the limited dataset is not suitable for estimation of reliable positivity rates.

Studies of SARS‐CoV‐2 outbreaks in nursing facilities showed a high effectiveness of a vaccination, preventing severe illness but not necessarily infection with SARS‐CoV‐2.^18^, ^19^ In contrast to reports on breakthrough infections after completed vaccinations,^20^, ^21^ in this single‐center retrospective study the elderly vaccinees already profited from a partial vaccination and development of severe COVID19 symptoms was restricted to the unvaccinated staff members.

The detection of antibodies to SARS‐CoV‐2 nucleocapsid and spike protein 3 months after putative exposure confirms a genuine viral infection despite a single vaccination. Missing an adequate humoral immune response in one SARS‐CoV‐2 RNA positive person might be caused by an inefficient immune system of the 92 years old resident. Humoral immune response 3 weeks after putative exposure was seen in only one‐third of the serum samples suggesting an incomplete protection at that early time point. Cellular immunity as an important part of the immune defense to corona viruses could not be determined due to missing blood samples.

Taken together, application of a single vaccine dose already provides a benefit in the elderly to prevent a severe COVID 19 disease. Nevertheless, the data also demonstrate that subsequent monitoring and adherence to the personal protection measures are strictly required, as the presence of SARS‐CoV‐2 RNA in oropharyngeal swabs of the asymptomatic individuals suggests that a transmission is still possible.

## CONFLICT OF INTERESTS

The authors declare that there are no conflict of interests.

## AUTHOR CONTRIBUTIONS

Rolf Schwarzer, Peter Menzel, Jörg Hofmann wrote the first draft. Sabine D.Freys was involved in patient care and collection of samples. Rolf Schwarzer, Nick Neuwinger, Nina Beikert, Bettina Eberspächer, Anke Edelmann, Marta Zuchowski, Inga Slothouwer, Angela Stein, Kathrin Theil, Peter Menzel, and Jörg Hofmann were involved in collection of samples and routine diagnostic testing. Rolf Schwarzer, Anke Edelmann, Kathrin Theil, and Peter Menzel contributed SARS‐CoV‐2 testing, sequencing, and phylogeny. Rolf Schwarzer and Jörg Hofmann  conceived, designed, and coordinated the study.

## ETHICS STATEMENT

Written informed consent is available for all senior residents and staff members included in this report.

## Supporting information

Supporting information.

Supporting information.
