# Supplementary material for: A single dose of the Biontech/Pfizer BNT162b2 vaccine protected elderly residents from severe COVID‐19 during a SARS‐coronavirus‐2 outbreak in a senior citizen home in Germany
Source: Immun Inflamm Dis. 2021 Sep 16;9(4):1809–14. doi: 10.1002/iid3.532 (PMC8589371; doi:10.1002/iid3.532)
Supplement: Supplementary file 1 — Supporting information. [file IID3-9-1809-s001.pdf]

We gratefully acknowledge the following Authors from the Originating laboratories responsible for obtaining the specimens, as well as the Submitting laboratories where the genome data were generated and shared via GISAID, on which this research is based.

All Submitters of data may be contacted directly via www.gisaid.org

Authors are sorted alphabetically.

Accession ID	Originating Laboratory	Submitting Laboratory	Authors
EPI_ISL_1140667, EPI_ISL_1140724, EPI_ISL_1140754, EPI_ISL_1140759, EPI_ISL_1140795, EPI_ISL_1142276, EPI_ISL_1142280, EPI_ISL_1142283, EPI_ISL_1142289, EPI_ISL_1142290, EPI_ISL_1142291, EPI_ISL_1142294, EPI_ISL_1142296, EPI_ISL_1142298, EPI_ISL_1142301, EPI_ISL_1142302, EPI_ISL_1142309, EPI_ISL_1142313, EPI_ISL_1142318, EPI_ISL_1142320, EPI_ISL_1142322, EPI_ISL_1142338, EPI_ISL_1142345, EPI_ISL_1142353, EPI_ISL_1142358, EPI_ISL_1142365, EPI_ISL_1142368, EPI_ISL_1142371, EPI_ISL_1142372, EPI_ISL_1142375, EPI_ISL_1142381, EPI_ISL_1142382, EPI_ISL_1142384, EPI_ISL_1142392, EPI_ISL_1142393, EPI_ISL_1142396, EPI_ISL_1142397, EPI_ISL_1142400, EPI_ISL_1142403, EPI_ISL_1142406, EPI_ISL_1142410, EPI_ISL_1142413, EPI_ISL_1142414, EPI_ISL_1142416, EPI_ISL_1142430, EPI_ISL_1142435, EPI_ISL_1142447, EPI_ISL_1142453, EPI_ISL_1142456, EPI_ISL_1142457, EPI_ISL_1142458, EPI_ISL_1142460, EPI_ISL_1142462, EPI_ISL_1142464, EPI_ISL_1142468, EPI_ISL_1142480, EPI_ISL_1142481, EPI_ISL_1142484, EPI_ISL_1142486, EPI_ISL_1142488, EPI_ISL_1142492, EPI_ISL_1142495, EPI_ISL_1142499, EPI_ISL_1142503, EPI_ISL_1142506, EPI_ISL_1142511, EPI_ISL_1142515, EPI_ISL_1142517, EPI_ISL_1142520, EPI_ISL_1142522, EPI_ISL_1142721, EPI_ISL_1142724, EPI_ISL_1142728, EPI_ISL_1142729, EPI_ISL_1142732, EPI_ISL_1142734, EPI_ISL_1142739, EPI_ISL_1142741, EPI_ISL_1142742, EPI_ISL_1142743, EPI_ISL_1142752, EPI_ISL_1142754, EPI_ISL_1142755, EPI_ISL_1142764, EPI_ISL_1142765, EPI_ISL_1142768, EPI_ISL_1142771, EPI_ISL_1142776, EPI_ISL_1142778, EPI_ISL_1142779, EPI_ISL_1142782, EPI_ISL_1142784, EPI_ISL_1142787, EPI_ISL_1142788, EPI_ISL_1142789, EPI_ISL_1142792, EPI_ISL_1142793, EPI_ISL_1142798, EPI_ISL_1142799, EPI_ISL_1142800, EPI_ISL_1142801, EPI_ISL_1142802, EPI_ISL_1142803, EPI_ISL_1142812, EPI_ISL_1142820, EPI_ISL_1142821, EPI_ISL_1142825, EPI_ISL_1142826, EPI_ISL_1142828, EPI_ISL_1142830, EPI_ISL_1142834, EPI_ISL_1142836, EPI_ISL_1142837, EPI_ISL_1142839, EPI_ISL_1142840, EPI_ISL_1142841, EPI_ISL_1142844, EPI_ISL_1142845, EPI_ISL_1142851, EPI_ISL_1142853, EPI_ISL_1142854, EPI_ISL_1142858, EPI_ISL_1142859, EPI_ISL_1142860, EPI_ISL_1142861, EPI_ISL_1142862, EPI_ISL_1142867, EPI_ISL_1142868, EPI_ISL_1142869, EPI_ISL_1142871, EPI_ISL_1142874, EPI_ISL_1142877, EPI_ISL_1142880, EPI_ISL_1142884, EPI_ISL_1142885, EPI_ISL_1142887, EPI_ISL_1142888, EPI_ISL_1142891, EPI_ISL_1142898, EPI_ISL_1142900, EPI_ISL_1142903, EPI_ISL_1142906, EPI_ISL_1142909, EPI_ISL_1142911, EPI_ISL_1142913, EPI_ISL_1142916, EPI_ISL_1142917, EPI_ISL_1142919, EPI_ISL_1142922, EPI_ISL_1142926, EPI_ISL_1142928, EPI_ISL_1142932, EPI_ISL_1142933, EPI_ISL_1142935, EPI_ISL_1142937, EPI_ISL_1142940, EPI_ISL_1142945, EPI_ISL_1142951, EPI_ISL_1142952, EPI_ISL_1142955, EPI_ISL_1142958, EPI_ISL_1142962, EPI_ISL_1142964, EPI_ISL_1142966, EPI_ISL_1142970, EPI_ISL_1142977, EPI_ISL_1142983, EPI_ISL_1142985, EPI_ISL_1142986, EPI_ISL_1142988, EPI_ISL_1142990, EPI_ISL_1142991, EPI_ISL_1142994, EPI_ISL_1142995, EPI_ISL_1143000, EPI_ISL_1143003, EPI_ISL_1143004, EPI_ISL_1143006, EPI_ISL_1143007, EPI_ISL_1143008, EPI_ISL_1143011, EPI_ISL_1143012, EPI_ISL_1143014, EPI_ISL_1143020, EPI_ISL_1143027, EPI_ISL_1143089, EPI_ISL_1143097, EPI_ISL_1143102, EPI_ISL_1143107, EPI_ISL_1143118, EPI_ISL_1143132, EPI_ISL_1143161, EPI_ISL_1143206, EPI_ISL_1143243, EPI_ISL_1143298, EPI_ISL_1145187			
see above	Sonic - MVZ Medizinisches Labor Bremen GmbH	Robert Koch Institute	unknown
EPI_ISL_860786	Charité Universitätsmedizin Berlin, Institute of Virology, Charitéplatz 1, 10117 Berlin, Germany	Charité Universitätsmedizin Berlin, Institute of Virology, Charitéplatz 1, 10117 Berlin, Germany	Victor M Corman, Julia Schneider, Jörn Beheim-Schwarzbach, Tobias Bleicker, Julia Tesch, Barbara Mühlemann, Talitha Veith, Terry Jones, Christian Drosten
EPI_ISL_862153, EPI_ISL_862154, EPI_ISL_862155, EPI_ISL_862156	Charité Universitätsmedizin Berlin, Institut fur Virologie/Labor Berlin	Charité Universitätsmedizin Berlin, Institut für Virologie	Victor M Corman, Barbara Mühlemann, Jörn Beheim-Schwarzbach, Tobias Bleicker, Julia Tesch, Talitha Veith, Julia Schneider, Terry Jones, Christian Drosten
EPI_ISL_909747	Charité Universitätsmedizin Berlin, Institut für Virologie/Labor Berlin	Charité Universitätsmedizin Berlin, Institut für Virologie	Victor M Corman, Barbara Mühlemann, Jörn Beheim-Schwarzbach, Tobias Bleicker, Julia Tesch, Talitha Veith, Julia Schneider, Terry Jones, Christian Drosten
