# Supplementary material for: A single dose of the Biontech/Pfizer BNT162b2 vaccine protected elderly residents from severe COVID‐19 during a SARS‐coronavirus‐2 outbreak in a senior citizen home in Germany
Source: Immun Inflamm Dis. 2021 Sep 16;9(4):1809–14. doi: 10.1002/iid3.532 (PMC8589371; doi:10.1002/iid3.532)
Supplement: Supplementary file 2 — Supporting information. [file IID3-9-1809-s002.pdf]

A Single dose of the Biontech/Pfizer BNT162b2 vaccine protected elderly residents from severe COVID-19 during a SARS-Coronavirus-2 outbreak in a senior citizen home in Germany

R. Schwarzer, S. D. Freys, N. Neuwinger, N. Beikert, B. Eberspaecher, A. Edelmann, M. Zuchowski, I. Slothouwer, A. Stein, K. Theil, P. Menzel, and J. Hofmann

Supplementary Figures

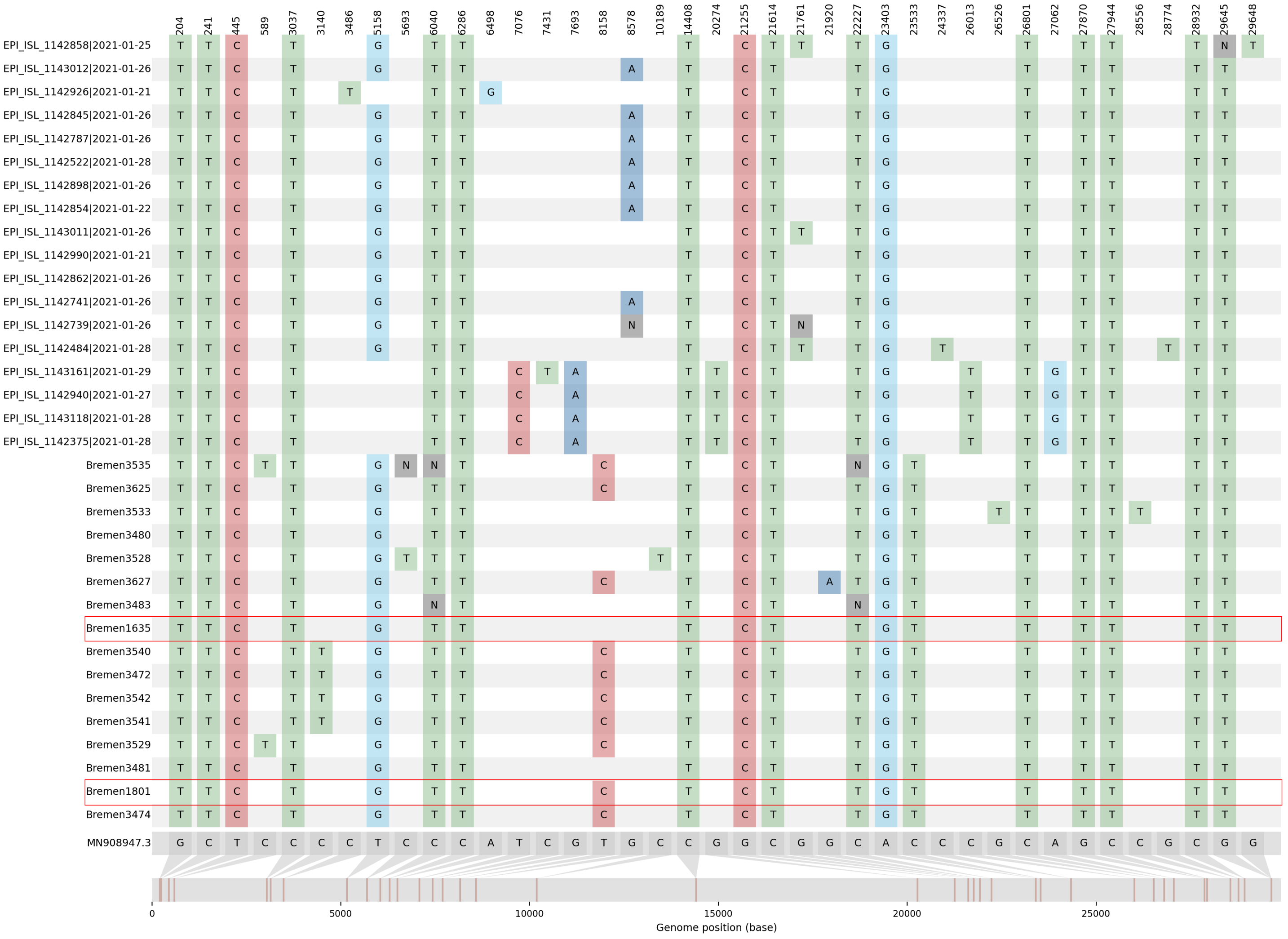

Suppl. Fig. S1: SNP positions in the whole genome alignment of SARS-CoV-2 genome sequences of the 14 residents and two staff members (red outline) from this study (labeled as Bremen…) as well as 18 sequences downloaded from GISAID that were collected in Bremen in January 2021 and belong to the PANGO lineage B.1.177.86. The SNP C23533T is found exclusively in all of the 16 sequences from this study
